# Eosinophilic and type 2 inflammation in bronchiectasis: from phenotype recognition toward endotype-informed therapy

**DOI:** 10.3389/fmed.2026.1987219

**Published:** 2026-10-01

**Authors:** Luwen Liang, Ling Liu, Caoqing Huang, Zhiping Zeng, Yunya Zhu

**Affiliations:** 1 Department of General Practice, The First Affiliated Hospital of Gannan Medical University, Ganzhou, China; 2 Department of Respiratory Medicine, Meizhou People’s Hospital, Meizhou, Guangdong, China

**Keywords:** biologics, biomarkers, bronchiectasis, endotype, eosinophils, inhaled corticosteroids, precision medicine, type 2 inflammation

## Abstract

Bronchiectasis is increasingly recognized as biologically heterogeneous, yet the clinical meaning of eosinophilic and type 2 (T2)-associated signals remains uncertain. This review reassesses whether the field has progressed from recognition of an eosinophilic phenotype to a bronchiectasis-specific, therapeutically actionable T2 endotype. Blood eosinophil count (BEC), airway eosinophilia, fractional exhaled nitric oxide, total immunoglobulin E, and pathway-level signatures capture overlapping but non-equivalent features. Current evidence supports an eosinophilic phenotype more strongly than a unified bronchiectasis-specific T2 endotype. Airway studies confirm eosinophil activation in selected patients, but direct evidence for a complete and reproducible epithelial alarmin–T2-cell–IL-4/IL-5/IL-13 pathway is limited, and asthma, allergic bronchopulmonary aspergillosis, fungal sensitization, chronic rhinosinusitis, and chronic obstructive pulmonary disease can confound biomarker interpretation. Prognostic associations are non-linear and context dependent: persistent or high eosinophilia and very low BEC have each been associated with adverse outcomes in different settings. Inhaled corticosteroids show biological effects and exploratory eosinophil-associated response signals, but these do not validate BEC as a predictive biomarker or support treatment solely for elevated BEC. In MAHALE, benralizumab produced profound eosinophil depletion without demonstrated clinical efficacy in the population studied; severe under-recruitment and limited eosinophilic enrichment restrict inference for persistently eosinophilic disease. Evidence from biologic-treated severe asthma with coexisting bronchiectasis remains indirect for bronchiectasis efficacy. The review therefore defines the validation pathway required before phenotype recognition can inform treatment selection: repeated stable-state phenotyping, airway biological validation, separation from T2 comorbidities, prespecified treatment-by-biomarker interaction analyses, and adequately powered biomarker-enriched trials.

## 1 Introduction

Bronchiectasis is a chronic, heterogeneous airway disease characterized by irreversible bronchial dilatation, impaired mucociliary clearance, recurrent respiratory symptoms, exacerbations, and susceptibility to chronic airway infection. Airway inflammation is central to disease activity, and the dominant biological framework has historically been neutrophilic. Sputum neutrophil elastase activity is associated with greater disease severity, exacerbation risk, and lung-function decline, while neutrophil extracellular trap (NET)-associated inflammation has been linked to impaired quality of life, future hospitalization, and mortality (1, 2). These findings establish neutrophilic inflammation as a major disease axis, but they do not fully explain the biological heterogeneity now apparent across contemporary bronchiectasis cohorts (3–5).

A reproducible eosinophilic phenotype has emerged within this broader inflammatory landscape. In a European multicohort analysis, approximately one fifth of patients met a blood eosinophil count (BEC) threshold of ≥300 cells/µL after exclusion of physician-diagnosed asthma and active allergic bronchopulmonary aspergillosis (ABPA) (6). Airway-focused studies have subsequently demonstrated sputum eosinophilia and eosinophil-derived proteins in selected patients in whom overt asthma was excluded, supporting the existence of eosinophilic inflammation beyond conventional asthma-bronchiectasis overlap (7, 8). Registry data also identify eosinophilic bronchiectasis in the absence of recognized Aspergillus-associated lung disease (9).

The biological interpretation of this signal remains unsettled. Peripheral eosinophilia, airway eosinophilia, and a mechanistically coherent type 2 endotype are not interchangeable constructs. Blood and airway eosinophilic measures show only modest concordance, different type 2-associated biomarkers classify partially overlapping patient groups, and BEC categories are incompletely stable over time (8, 10–13). Accordingly, an elevated BEC is best viewed as an accessible eosinophilic phenotype marker rather than sufficient evidence of a bronchiectasis-specific type 2 endotype.

This distinction has direct therapeutic implications. Secondary analyses of inhaled corticosteroid (ICS) trials and large observational cohorts suggest that eosinophilia may modify corticosteroid response, but these signals remain unconfirmed in a prospective biomarker-stratified trial (14–16). The MAHALE randomized trial added a critical test of the endotype hypothesis: benralizumab produced profound eosinophil depletion but did not demonstrate a reduction in bronchiectasis exacerbations or improvement in major clinical outcomes; interpretation was limited by marked under-recruitment and limited eosinophilic enrichment (17). Thus, target engagement alone cannot validate a clinically actionable endotype.

The central question is no longer whether eosinophilic inflammation occurs in bronchiectasis, but what evidence is required before it can be called a therapeutically actionable, bronchiectasis-specific T2 endotype. Existing reviews describe eosinophilic signals, inflammatory heterogeneity, and emerging therapies; this review instead follows the evidentiary sequence from phenotype recognition through temporal reproducibility, airway biological validation, comorbidity independence, clinical relevance, and prospective treatment interaction (18, 19). It distinguishes eosinophilic phenotype, airway eosinophilia, T2-associated inflammation, candidate endotype, and clinically actionable endotype, and identifies the trial design needed to bridge those categories (20–24).

## 2 Beyond the neutrophilic paradigm: inflammatory heterogeneity in bronchiectasis

Recognition of eosinophilic inflammation should not be interpreted as replacement of the neutrophilic model. Neutrophil-dominated inflammation remains one of the best validated biological features of bronchiectasis. Neutrophil elastase correlates with the Bronchiectasis Severity Index, airflow limitation, and radiological extent, and prospectively associates with exacerbation frequency and FEV1 decline (1). NET-related signatures extend this framework by associating with severity, quality of life, hospitalization, and mortality and by changing during successful antimicrobial or macrolide treatment (2, 25, 26).

Airway infection constitutes a second major dimension. Bronchiectasis harbours persistent polymicrobial communities, and lower diversity or Pseudomonas aeruginosa dominance is associated with more severe disease and adverse outcomes (27, 28). Yet microbiological and inflammatory states do not map onto a simple binary classification. Elevated BEC and airway eosinophilic signals can coexist with Pseudomonas-dominated microbiota or chronic infection (6, 8). Eosinophilic bronchiectasis should therefore not be equated with a non-infectious counterpart of neutrophilic disease (5, 29).

Molecular endotyping provides stronger evidence for overlapping inflammatory programs. In a multicentre European cohort, profiling of serum and sputum mediators identified several inflammatory clusters, including mixed neutrophilic/type 2-associated states that differed in microbiome composition and future exacerbation risk (30). Smaller induced-sputum studies similarly identify eosinophilic and mixed granulocytic phenotypes (31). These observations support simultaneous activation of partially independent inflammatory pathways rather than mutually exclusive neutrophilic and eosinophilic subtypes (32).

Inflammatory classification is also state dependent. Molecular profiling of paired stable and exacerbation samples has identified bacterial, viral, eosinophilic, and other exacerbation endotypes (33). Together with longitudinal BEC instability (10, 11), these data caution against treating a single biological measurement as a permanent patient-level label.

The most defensible current model is therefore an overlapping inflammatory spectrum in which neutrophilic/infection-associated and eosinophilic/type 2-associated activity represent partially independent dimensions. This framework preserves the central role of infection and neutrophil biology while allowing a subset of patients to exhibit reproducible eosinophilic features that require separate biological and therapeutic evaluation (Figure 1) (34, 35).

**Figure 1:**
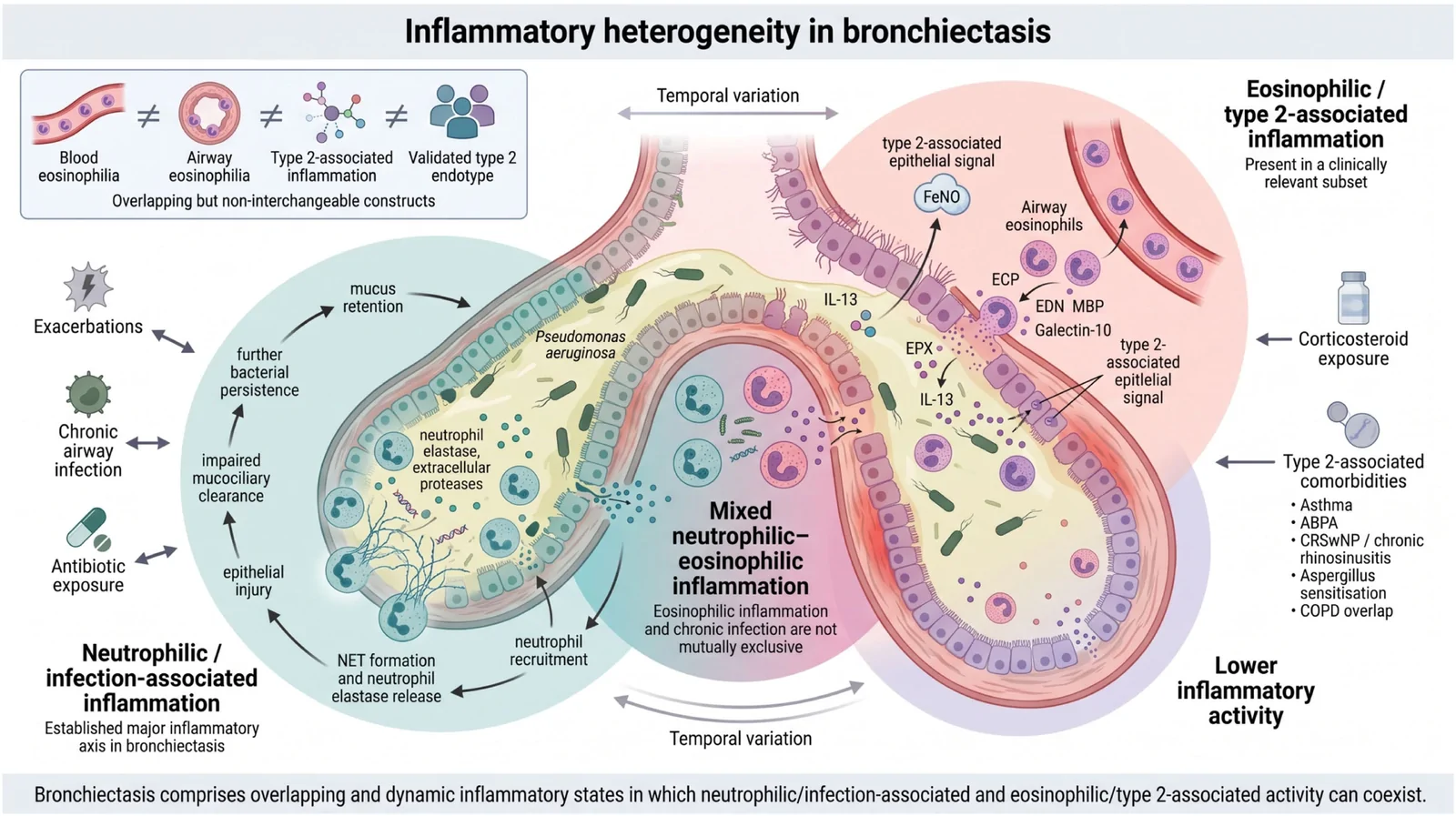
Inflammatory heterogeneity in bronchiectasis. Neutrophilic/infection-associated and eosinophilic/type 2-associated inflammatory activity are depicted as overlapping and dynamic dimensions. Blood eosinophilia, airway eosinophilia, and type 2-associated signals are related but non-equivalent, while infection, exacerbations, treatment exposure, and type 2-associated comorbidities may modify inflammatory state over time.

## 3 Defining eosinophilic and type 2 inflammation in bronchiectasis

The terminology surrounding eosinophilic and type 2 inflammation has developed faster than consensus definitions. A circulating eosinophilic phenotype, eosinophilic inflammation within the airway, a type 2-associated biomarker profile, and a true mechanistic endotype represent progressively stronger levels of biological inference. Distinguishing these constructs is essential to avoid overestimating prevalence and overinterpreting therapeutic signals (36).

BEC is currently the most accessible marker of eosinophilic inflammation, but it is a systemic surrogate rather than a direct measure of airway biology. Studies have used absolute thresholds ranging from approximately 100 to 300 cells/µL and percentage-based definitions, often for different purposes (6, 14, 15, 37, 38). The widely used ≥300 cells/µL threshold is useful for phenotypic stratification but has not been prospectively validated as a bronchiectasis-specific type 2 endotype threshold.

Airway eosinophilia is biologically more proximal. Induced or spontaneous sputum studies have identified eosinophilic and mixed granulocytic phenotypes, commonly using sputum eosinophils around >3% as an operational threshold (6, 7, 31, 39). However, blood and airway measures are only partially concordant. In selected idiopathic or post-infectious bronchiectasis, BEC provided only moderate discrimination for sputum eosinophilia (7). EMBARC-BRIDGE directly quantified sputum eosinophil-derived proteins and found only a modest relationship with circulating eosinophils (8). Earlier work also demonstrated eosinophil activation despite the absence of peripheral eosinophilia (40). These findings make it inappropriate to use BEC as a proxy for the magnitude of airway eosinophilic activity without qualification.

The term type 2 inflammation requires stronger biological evidence. Canonical type 2 biology encompasses coordinated signaling involving epithelial alarmins, ILC2 and/or Th2 responses, IL-4, IL-5, IL-13, eosinophil recruitment and activation, IgE-associated responses, and epithelial mucus programs. In bronchiectasis, direct evidence for this complete pathway is limited. Small airway studies link sputum eosinophilia with FeNO and IL-13 (31), but this does not establish that all eosinophilic bronchiectasis shares a uniform Th2/ILC2 mechanism.

Discordance among BEC, FeNO, and total IgE reinforces this point. Prospective studies excluding asthma and ABPA show that these markers identify substantially different patient groups (12, 13). A single-centre cohort reported an eosinophilic phenotype in 27% using BEC ≥ 300 cells/µL but an operational BEC-plus-FeNO type 2-associated phenotype in approximately 20%; this composite definition was investigator-defined rather than validated against airway molecular biology or treatment response (41). Broad OR-rule composites can therefore inflate apparent prevalence by pooling biologically distinct signals.

Temporal reproducibility adds another requirement. Repeated stable-state measurements show reasonable short-term BEC reliability but increasing movement between categories over longer follow-up, and only a subset of patients with baseline eosinophilia remain persistently eosinophilic (10, 11). A single stable-state measurement therefore cannot be assumed to define a durable endotype.

For this review, blood eosinophilia refers to an elevated circulating eosinophil count according to the threshold used in the relevant study; airway eosinophilia refers to direct evidence from sputum cells or eosinophil-derived proteins; a type 2-associated biomarker profile denotes concordant markers compatible with type 2 biology after consideration of competing diagnoses; and a candidate type 2 endotype implies a reproducible, mechanistically coherent state with potential relevance to differential treatment response. Current evidence is strongest for the eosinophilic phenotype and substantially weaker for the final endotype category (Table 1).

**Table 1:** Proposed terminology for eosinophilic and type 2-associated inflammation in bronchiectasis.

Term	Operational definition	Typical measurements/criteria	Biological interpretation	Current status	Key caveat	Key refs
Blood eosinophilia	Elevation of circulating eosinophils above a prespecified study threshold.	Absolute BEC, commonly ≥ 300 cells/µL; lower absolute or percentage thresholds have also been used.	Accessible systemic eosinophilic phenotype; not a direct measure of airway inflammation.	Clinically feasible phenotyping marker.	No universally validated bronchiectasis-specific threshold; values vary over time and show only modest airway concordance.	(6, 10–13, 38, 41)
Airway eosinophilia	Direct evidence of increased eosinophils within bronchiectatic airways.	Sputum eosinophil differential count, often using an operational threshold around >3%.	More proximal measure of airway eosinophilic inflammation.	Mechanistically informative; specialist/research use.	Sampling and processing are less standardized, and thresholds have not been prospectively validated for treatment selection.	(6–8, 31, 39)
Eosinophil activation	Evidence that eosinophils are activated or degranulating within the airway, regardless of cell abundance.	ECP, EPX, EDN, MBP, galectin-10 and related eosinophil-derived proteins.	Functional eosinophil activity rather than cell count alone.	Research biomarker.	Specialized assays; limited longitudinal and treatment-response validation.	(8, 40)
Type 2-associated biomarker profile	Concordant or partially concordant biomarkers compatible with type 2 biology.	BEC, FeNO, total IgE, airway eosinophils, eosinophil proteins and selected cytokine signals.	Suggests type 2-associated biology but does not prove one unified mechanism.	Exploratory/research framework.	BEC, FeNO and IgE are frequently discordant; broad OR-rule composites can inflate apparent prevalence.	(12, 13, 31, 41)
Eosinophilic phenotype	Reproducible systemic and/or airway eosinophilic characteristic in bronchiectasis.	Preferably repeated stable-state BEC, with airway confirmation when available.	Clinical phenotype that may capture several underlying mechanisms.	Supported by multiple bronchiectasis cohorts.	Persistence is incomplete, and comorbid asthma, ABPA, CRS/CRSwNP and COPD overlap require characterization.	(6, 10, 11, 41, 79, 80)
Candidate type 2 endotype	A reproducible, mechanistically coherent type 2-associated biological state attributable to bronchiectasis rather than a competing comorbidity.	Longitudinal biomarkers plus airway biological confirmation and, ideally, molecular/pathway evidence.	Candidate disease mechanism rather than a purely descriptive phenotype.	Not yet validated.	A complete bronchiectasis-specific epithelial alarmin–ILC2/Th2–IL-4/IL-5/IL-13 pathway has not been reproducibly established.	(8, 30, 31)
Clinically actionable type 2 endotype	A validated endotype that identifies differential clinical benefit from a targeted therapy.	Reproducible endotype plus prespecified treatment-by-biomarker/endotype interaction in prospective trials.	Predictive biological subtype suitable for treatment selection.	Not established in bronchiectasis.	Prognostic association and pharmacological target engagement are insufficient without prospective evidence of differential treatment response.	(14–17, 60)

Proposed terminology is intended to distinguish phenotype recognition from mechanistic and predictive endotyping. No single biomarker currently defines a validated bronchiectasis-specific type 2 endotype. ABPA, allergic bronchopulmonary aspergillosis; BEC, blood eosinophil count; COPD, chronic obstructive pulmonary disease; CRS, chronic rhinosinusitis; CRSwNP, chronic rhinosinusitis with nasal polyps; ECP, eosinophil cationic protein; EDN, eosinophil-derived neurotoxin; EPX, eosinophil peroxidase; FeNO, fractional exhaled nitric oxide; MBP, major basic protein.

## 4 Prevalence and temporal stability of the eosinophilic phenotype

The prevalence of eosinophilic or type 2-associated inflammation varies substantially across studies because the operational definitions are not equivalent. When BEC ≥ 300 cells/µL is used in cohorts that exclude overt asthma and ABPA, approximately 15%–25% of patients are generally classified as eosinophilic. Shoemark and colleagues reported 22.6% in a large European multicohort analysis (6), while another prospective cohort reported approximately 15% (12). A single-centre study reported 27%, although COPD remained common and therefore represented residual overlap confounding (41). These data support a meaningful eosinophilic subgroup but not a universal prevalence estimate.

Prevalence changes when other biomarkers are used. FeNO and total IgE identify partly overlapping populations, and composite definitions vary markedly according to whether concordant positivity is required, or any single positive marker is sufficient (12, 13, 41). Thus, a high prevalence generated by a broad type 2 biomarker definition should not be interpreted as evidence that the same proportion of patients has a unified mechanistic endotype.

The sampled biological compartment further contributes to variability. Peripheral blood eosinophilia, sputum eosinophilia, and airway eosinophil-derived proteins are only partially concordant (6–8). Studies also differ in whether asthma, ABPA, Aspergillus sensitisation, COPD, chronic rhinosinusitis, or nasal polyposis are excluded, adjusted for, or simply recorded. Corticosteroid exposure, recent infection, and timing relative to exacerbation provide additional sources of variation.

Single-time-point estimates also assume phenotype stability. Longitudinal RIBRON data show increasing movement between BEC categories with longer follow-up, and persistent eosinophilia is present in only a proportion of patients classified as eosinophilic at baseline (10, 11). Acute eosinophilic exacerbations represent a related but non-equivalent construct: hospitalized cohorts and molecular profiling identify eosinophilic exacerbation states (33, 39, 42), but these should not be used to infer a stable baseline endotype.

The most defensible summary is therefore that eosinophilic inflammation occurs in a meaningful minority of patients, while its estimated prevalence and persistence are highly dependent on threshold, compartment, disease state, treatment exposure, and handling of type 2 comorbidities. Repeated stable-state assessment is likely to be more informative for endotype research than a single baseline BEC, although the optimal number and timing of measurements remain undefined.

## 5 Immunobiology of type 2-associated inflammation in bronchiectasis

The biological basis of eosinophilic inflammation in bronchiectasis is less mature than its clinical phenotype. Direct evidence supports airway eosinophil accumulation and activation and selected type 2-associated signals, but the complete epithelial alarmin-ILC2/Th2-IL-4/IL-5/IL-13 cascade established in asthma has not been demonstrated with comparable depth in bronchiectasis (43).

Eosinophil activation can occur independently of peripheral eosinophilia. An early non-CF, non-ABPA study demonstrated increased eosinophil cationic protein without increased circulating eosinophil counts (40). More recently, sputum differential counts and direct proteomic quantification of eosinophil peroxidase, eosinophil-derived neurotoxin, eosinophil cationic protein, major basic protein, and galectin-10 have confirmed eosinophil activity within bronchiectatic airways (8).

Bronchiectasis-specific evidence for canonical type 2 cytokines is limited. A small induced-sputum study found that an eosinophilic airway phenotype was associated with higher FeNO and sputum IL-13 (31). This is biologically compatible with type 2 epithelial signaling but insufficient to establish a uniform IL-13-driven mechanism. The functional relevance of the IL-5 axis is supported by the ability of benralizumab to deplete blood and sputum eosinophils in MAHALE (17), yet the absence of corresponding clinical efficacy shows that pharmacological suppression of eosinophils does not by itself identify a disease-driving pathway.

Evidence for upstream epithelial alarmins such as TSLP, IL-33, and IL-25 and for IL-4/IL-13-mediated remodeling remains largely extrapolated from asthma and other eosinophilic airway diseases. These pathways are biologically plausible candidates in bronchiectasis but should be represented as incompletely demonstrated rather than established mechanisms. The same caution applies to type 2-driven mucus biology: mucus retention is central to bronchiectasis, but a causal IL-13-driven mucus program has not yet been demonstrated as a defining mechanism in bronchiectasis-specific cohorts.

Importantly, eosinophilic and neutrophilic pathways can be simultaneously active. Cellular and molecular studies identify mixed granulocytic and mixed neutrophilic/type 2-associated states (30, 31). ABPA provides an instructive but etiologically distinct example in which eosinophilic and neutrophilic airway inflammation coexist (44). ABPA data therefore support the plausibility of mixed inflammation but should not be used to infer the mechanism of bronchiectasis without fungal allergic disease.

The mechanistic evidence thus supports a limited model: direct airway eosinophil activation is established, selected type 2-associated signals are present in some patients, and these signals can coexist with neutrophilic inflammation. A complete, reproducible bronchiectasis-specific type 2 pathway remains to be established (Figure 2).

**Figure 2:**
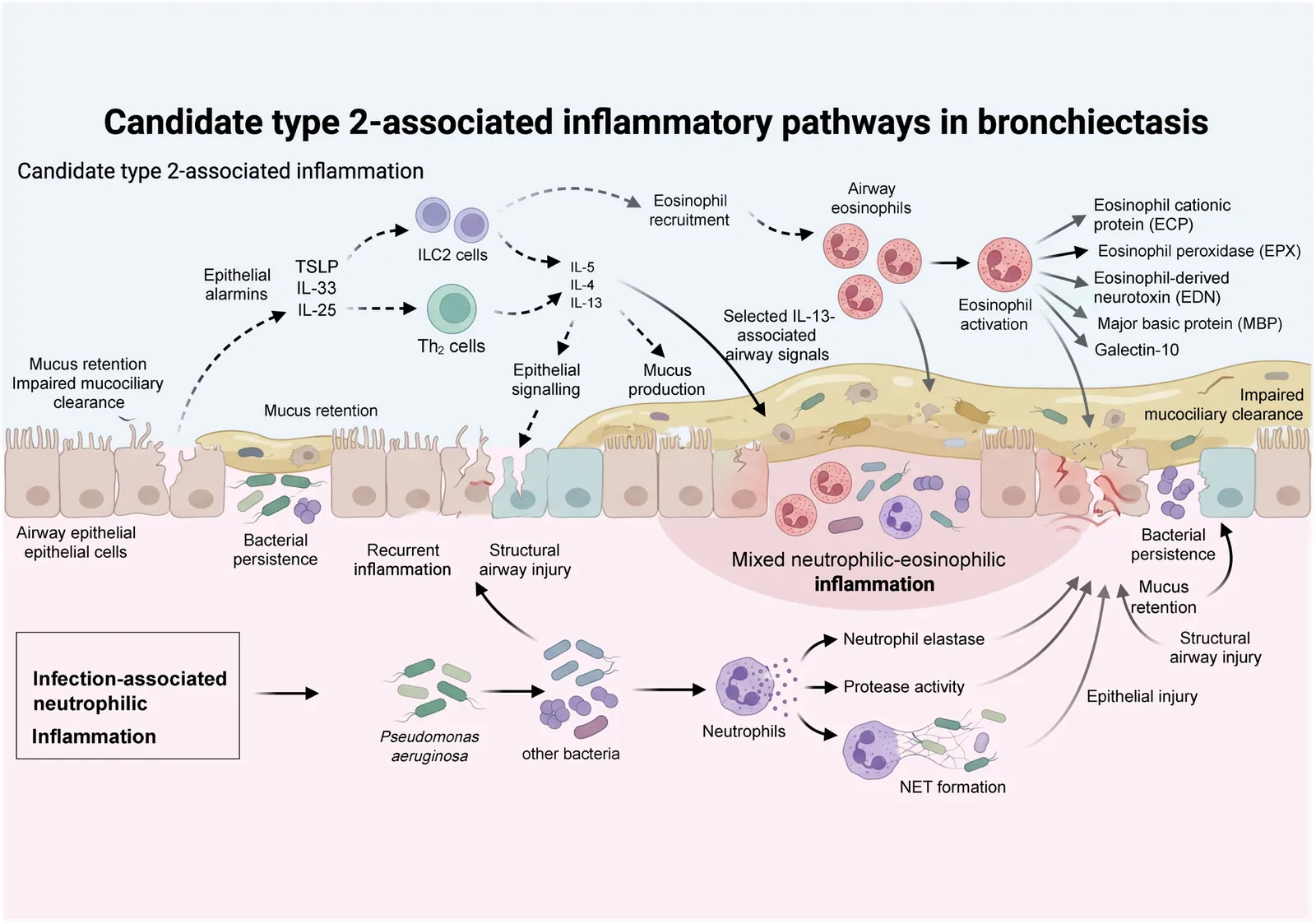
Candidate type 2-associated inflammatory pathways in bronchiectasis. The schematic integrates direct evidence of airway eosinophil accumulation and activation with candidate type 2-associated pathways. Solid elements indicate observations supported directly in bronchiectasis, whereas dashed or contextual elements denote mechanistically plausible pathways that remain incompletely validated and are partly extrapolated from asthma or other eosinophilic airway diseases.

## 6 Type 2-associated inflammation and the airway microbiome

Chronic airway infection and microbial dysbiosis are central to bronchiectasis pathophysiology. Lower bacterial diversity and Pseudomonas dominance are associated with greater disease severity, exacerbation burden, and mortality (27, 28). The emergence of an eosinophilic phenotype therefore raises the question of whether it maps to a distinct microbiological state.

Available evidence argues against a simple separation. Elevated BEC can occur in both Streptococcus- and Pseudomonas-dominated microbiota (6), and EMBARC-BRIDGE detected eosinophil-derived proteins in airways that also harboured bacterial pathogens (8). A single-centre cohort likewise found chronic Pseudomonas colonization associated with a BEC-defined eosinophilic phenotype, although COPD remained a residual confounder (41). These studies collectively refute a sterile-eosinophilic vs. infected-neutrophilic dichotomy.

Integrated molecular studies reinforce this interpretation. Inflammatory clusters containing type 2-associated signals differed in microbial composition, yet mixed neutrophilic/type 2 states were evident (30). Microbiological and immunological classifications therefore describe interacting but non-equivalent dimensions of disease.

Fungal sensitisation adds a distinct layer. EMBARC data show clinically important ABPA, Aspergillus sensitisation, and eosinophilic bronchiectasis without recognized Aspergillus lung disease (9). A prospective multicentre study excluding known ABPA and primary asthma also identified Aspergillus fumigatus sensitisation as a high-risk phenotype, although occult ABPA could not be completely excluded (45). ABPA, Aspergillus sensitisation without ABPA, chronic fungal infection or colonization, and eosinophilic bronchiectasis without fungal disease should therefore be separated rather than pooled under a generic type 2 label (46–48).

Current observational studies cannot determine the direction of interaction between microbial communities and eosinophilic inflammation. Microbes may alter epithelial signaling and immune recruitment; eosinophilic inflammation may alter mucus or antimicrobial defense; or both may arise from shared determinants such as structural damage and impaired clearance (6, 8, 28, 30). A reproducible “type 2 microbiome” has therefore not been established. Future endotyping should integrate microbiology as an independent dimension rather than assume it is downstream of eosinophilic biology.

## 7 Biomarkers and endotyping

A clinically useful type 2 framework requires biomarkers that are reproducible, biologically meaningful, and relevant to treatment response. No current marker satisfies all three requirements. BEC offers the greatest feasibility; airway eosinophils and eosinophil-derived proteins provide greater biological proximity; FeNO and IgE provide complementary but nonspecific information; and multi-omic signatures remain developmental.

BEC is inexpensive, widely available, analytically standardized, and scalable for longitudinal assessment and trial enrichment (6, 10, 11, 38). Its limitations are equally important: it is not airway-specific, changes over time, and correlates only modestly with airway eosinophilic measures (8, 10). BEC should therefore be considered a practical eosinophilic stratifier rather than a validated endotype biomarker. Its predictive status is also unresolved. Positive ICS effect-modification signals coexist with negative replication and with the clinically negative, underpowered MAHALE trial (14, 16, 17, 49).

Sputum eosinophils provide a more proximal cellular phenotype and are associated with FeNO or IL-13 in selected cohorts (7, 31, 39). However, sputum induction, processing, quality criteria, and thresholds are not standardized for routine bronchiectasis care, and longitudinal treatment-predictive data are lacking. Eosinophil-derived airway proteins may capture activation more directly than cell counts, but currently require specialized assays and lack validated clinical cutoffs (8).

FeNO is attractive because it is non-invasive and reflects IL-4/IL-13-associated epithelial signaling in asthma. In bronchiectasis, however, FeNO and BEC are frequently discordant (12, 13, 41). An older randomized ICS study also found that exhaled nitric oxide did not behave as a consistent treatment-responsive biomarker (50). FeNO may therefore add contextual information but should not be treated as a standalone bronchiectasis type 2 test.

Total IgE and Aspergillus-specific biomarkers have a different role. Total IgE is nonspecific and is more useful in etiological evaluation for atopy and ABPA than in defining bronchiectasis-intrinsic type 2 inflammation (9, 12). Aspergillus-specific IgE and IgG identify fungal sensitisation or fungal-associated disease and should be interpreted as etiological biomarkers rather than generic type 2 markers (9, 45).

Cytokine and multi-omic approaches offer greater mechanistic resolution. Sputum IL-13 and related type 2 signals are detectable in small studies (31), while molecular and proteomic profiling identifies coordinated inflammatory states that cannot be reduced to a single BEC threshold (8, 30, 33). These approaches are conceptually closer to endotyping but remain platform- and cohort-dependent and require external replication, longitudinal stability testing, and prospective treatment-interaction validation.

Composite biomarker strategies may ultimately improve specificity, particularly if they require concordance across compartments rather than positivity for any single marker. At present, however, OR-rule composites based on BEC, FeNO, and IgE can combine biologically different groups (12, 13). No single biomarker or composite currently meets all requirements for a clinically validated bronchiectasis type 2 endotyping tool (Table 2).

**Table 2:** Candidate biomarkers for eosinophilic and type 2-associated inflammation in bronchiectasis.

Biomarker	Compartment	Main strength	Major limitation	Longitudinal evidence	Airway specificity	Current role	Key refs
Blood eosinophil count (BEC)	Peripheral blood	Inexpensive, standardized, scalable and readily repeatable; strongest current epidemiological evidence.	Systemic surrogate with modest blood–airway concordance; threshold heterogeneity; affected by time, disease state and treatment.	Moderate; repeated-measure studies show incomplete long-term category stability.	Low–moderate	Best currently available clinical stratification marker; candidate effect modifier, not a validated predictive biomarker.	(6, 10, 11, 14–16, 38, 41)
Sputum eosinophils	Airway	Direct cellular assessment of airway eosinophilic inflammation.	Induction/processing variability; not obtainable in all patients; no validated treatment-selection threshold.	Limited	High	Mechanistic and specialist phenotyping; potential enrichment biomarker for future trials.	(6–8, 31, 39)
Eosinophil-derived proteins (ECP, EPX, EDN, MBP, galectin-10)	Airway sputum	Captures eosinophil activation/degranulation and may reveal activity not reflected by BEC.	Specialized assays, limited standardization and limited prospective outcome data.	Very limited	High	Endotype discovery and airway biological validation.	(8, 40)
Fractional exhaled nitric oxide (FeNO)	Exhaled airway signal	Non-invasive, standardized and biologically linked to IL-4/IL-13-associated epithelial signaling.	Substantial discordance with BEC; influenced by asthma, atopy, smoking and other airway factors.	Limited	Moderate	Complementary T2-associated marker; not a standalone bronchiectasis endotyping test.	(12, 13, 31, 41, 50)
Total IgE	Peripheral blood	Widely accessible and useful in broader etiological evaluation.	Nonspecific; frequently discordant with BEC and FeNO.	Limited	Low	Primarily supports assessment for atopy/ABPA rather than generic T2 endotyping.	(9, 12, 82)
Aspergillus-specific IgE/IgG and sensitisation testing	Peripheral blood/sensitisation assessment	Helps identify fungal-associated disease and prevents misclassification of ABPA or sensitisation as intrinsic bronchiectasis T2 inflammation.	Etiology-specific rather than a generic marker of bronchiectasis T2 biology.	Established for diagnostic assessment, not endotype stability.	Etiologic	ABPA and fungal-associated disease assessment.	(9, 45, 82)
IL-13 and other T2-associated cytokines	Airway ± blood	Potentially greater mechanistic specificity than surrogate clinical biomarkers.	Small studies, low concentrations, assay variability and limited disease-specific replication.	Very limited	Potentially high	Mechanistic research; insufficient for routine classification.	(31)
Multi-omic inflammatory signatures	Airway and/or systemic	Captures coordinated biological networks and mixed inflammatory states beyond single-marker thresholds.	Cost, platform dependence, cohort-specific clustering and limited external validation.	Limited	Potentially high	Endotype discovery; requires external, longitudinal and treatment-response validation.	(8, 30, 33)

No biomarker currently satisfies all requirements for a standalone bronchiectasis type 2 endotyping tool: analytical feasibility, longitudinal reproducibility, airway biological specificity, independence from major type 2 comorbidities, and prospective prediction of differential treatment response.

## 8 Clinical outcomes associated with eosinophilic/type 2-associated inflammation

The clinical significance of eosinophilic inflammation is not captured by a simple higher-is-worse model. Across cohorts, eosinophilic markers are associated with exacerbations, lung function, hospitalization, and disease severity, but the direction and magnitude of these relationships vary with threshold, persistence, infection burden, and disease state (51, 52).

RIBRON data provide the clearest evidence of non-linearity. The relationship between BEC and bronchiectasis severity showed a U-shaped pattern, while earlier analyses from the same registry associated low BEC with more severe disease and poorer lung function (37, 38). Other cohorts also link low stable-state BEC or eosinopenia with hospitalized exacerbations, hospitalization, and mortality (53, 54). These observations do not imply that eosinophils are protective; rather, very low counts may mark a severe infection-dominant or systemic-inflammatory state.

At the other end of the spectrum, high or persistent eosinophilia can also associate with clinical activity. Higher BEC was associated with shorter time to exacerbation in a *post-hoc* analysis from the European multicohort study (6), and persistent eosinophilia in longitudinal RIBRON data was associated with greater severity and exacerbation burden (11). A single-centre cohort also reported more frequent exacerbations among patients with BEC ≥ 300 cells/µL, although substantial COPD overlap limits generalizability (41).

Acute exacerbation studies add further state dependence. Hospitalized eosinophilic exacerbations have been associated with greater airflow obstruction, higher severity scores, longer hospital stay, or higher cost in some cohorts (39, 42). Molecular profiling identifies eosinophilic exacerbations alongside bacterial and viral endotypes (33), indicating that the inflammatory pattern during an acute event should not be assumed to represent the patient's stable-state biology.

Associations with lung function and composite severity are similarly inconsistent. Some studies link eosinophilic markers with worse obstruction or severity, whereas others associate lower BEC with poorer lung function (37–39, 41). Airway eosinophil proteins show only weak relationships with spirometric and radiological measures (8). Evidence for quality of life, mortality associated with high BEC, and structural disease progression remains comparatively sparse.

The most defensible interpretation is therefore a non-linear and context-dependent relationship. Very low eosinophil counts may mark severe infection-associated disease, while persistent eosinophilia may define a separate phenotype with greater exacerbation susceptibility. BEC should not be presented as a standalone linear prognostic biomarker without longitudinal, microbiological, and clinical context.

## 9 Therapeutic implications

The therapeutic evidence should be interpreted in three layers: effects of anti-inflammatory therapy in unselected bronchiectasis, exploratory biomarker-treatment interactions, and direct or indirect studies of type 2-targeted biologics. Biological target engagement, clinical efficacy, and predictive biomarker validation are distinct evidentiary steps.

### 9.1 Inhaled corticosteroids

Older randomized trials show that ICS can modify airway inflammation or symptoms but have inconsistent effects on bronchiectasis exacerbations and lung function. Short-term fluticasone reduced sputum inflammatory indices without clear spirometric benefit (55), while a 12-month trial improved sputum volume in some patients but not overall exacerbation frequency or FEV1 (56). Another randomized study reported symptomatic and quality-of-life improvement without consistent effects on exacerbations or lung function (57), and budesonide reduced sputum eosinophils without establishing meaningful primary clinical benefit (58). These trials demonstrate anti-inflammatory activity but do not establish routine efficacy in unselected bronchiectasis.

Secondary analyses raise the possibility of eosinophil-dependent corticosteroid responsiveness. A *post-hoc* fluticasone analysis suggested greater response at higher BEC, whereas a budesonide reanalysis did not reproduce the interaction (14, 49). A pooled *post-hoc* analysis of earlier trials and subsequent observational studies reported fewer exacerbations among eosinophilic ICS-treated patients (15, 16, 59). However, the relevant thresholds were retrospective or data-derived, older trial datasets overlap, and the large EMBARC analysis remained nonrandomized. BEC should therefore be described as a candidate effect modifier rather than a validated predictive biomarker.

Current ERS guidance advises against routine long-term ICS in bronchiectasis without another established indication and does not support initiating ICS solely because BEC is elevated (60). This conservative position also reflects safety. Comparative observational data associate chronic ICS exposure with more hospitalized respiratory infections than macrolide therapy (61), and chronic airway-disease studies show a dose-related association between ICS and pulmonary non-tuberculous mycobacterial infection (62). COPD-bronchiectasis overlap data further suggest that pneumonia risk and its relationship with ICS may vary according to eosinophil level, although these findings are not directly generalizable to bronchiectasis without COPD (63, 64).

### 9.2 Type 2-targeted biologics

Most early biologic evidence comes from severe eosinophilic asthma with coexisting bronchiectasis. Small case series and real-world cohorts report improvements in asthma control, oral corticosteroid exposure, and asthma exacerbations with mepolizumab or benralizumab (65–68). Dupilumab-treated severe-asthma cohorts similarly report benefit in patients who also have bronchiectasis (69), and a recent prospective pilot suggests that mepolizumab can reduce mucus-plug burden in severe eosinophilic asthma with bronchiectasis (70). These studies support treatment of the coexisting severe type 2 airway disease; they do not establish bronchiectasis-specific efficacy.

MAHALE is therefore pivotal because it directly tested benralizumab in bronchiectasis. Benralizumab achieved near-complete eosinophil depletion but did not reduce annualized exacerbations or improve symptoms, lung function, or health-related quality of life (17). The trial was nevertheless severely under-recruited relative to plan, and only a small subgroup had BEC ≥ 300 cells/µL. It is therefore inappropriate either to claim efficacy or to conclude that anti-IL-5R therapy has been definitively disproven in a rigorously enriched persistent eosinophilic population. The key inference is narrower: biological target engagement did not translate into demonstrated clinical benefit in the population studied.

Bronchiectasis-specific efficacy evidence for anti-IL-4R*α* and anti-TSLP strategies remains insufficient. Extrapolation from asthma is particularly problematic because chronic infection, structural airway damage, and mixed neutrophilic inflammation may alter the relevance of canonical type 2 pathways (71).

### 9.3 DPP-1 inhibition and mixed inflammation

Brensocatib inhibits DPP-1-dependent activation of neutrophil serine proteases and should therefore be interpreted as targeting the neutrophilic/protease component of mixed inflammation rather than the canonical type 2 pathway (72). In the phase 3 ASPEN trial, brensocatib reduced pulmonary exacerbations in an unselected bronchiectasis population (73). In a *post-hoc* WILLOW analysis, the estimates for time to first exacerbation were numerically favourable both in patients with blood eosinophil counts ≥ 300 cells/µL (hazard ratio 0.49, 95% CI: 0.20–1.20) and in those with lower counts (0.66, 0.42–1.06); patients with elevated eosinophils had similar baseline sputum neutrophil elastase activity (74). These findings suggest that coexisting eosinophilia does not preclude benefit from DPP-1 inhibition. They do not, however, establish suppression of type 2 inflammation, a differential treatment effect by eosinophil status, or benefit from combined neutrophil- and type 2-directed treatment, because the subgroup analysis was *post hoc* and not powered for interaction testing, and published trials have not established longitudinal effects on FeNO, IgE, airway eosinophilia, or type 2 cytokines. Any indirect reduction in epithelial injury or infection-related type 2 signalling therefore remains a hypothesis requiring prospectively phenotyped studies.

Standard bronchiectasis management therefore remains foundational. Airway clearance, treatment of underlying causes, infection-directed strategies, and evidence-based long-term antibiotic therapy should not be displaced by an unvalidated type 2 framework. The therapeutic priority is not broader empirical use of corticosteroids or biologics, but prospective demonstration that a reproducible biomarker-defined subgroup derives differential bronchiectasis-specific benefit (Table 3) (75).

**Table 3:** Evidence for type 2-directed therapeutic strategies in bronchiectasis.

Therapeutic strategy	Target/rationale	Evidence base	Key finding	Biomarker signal	Major limitation	Current interpretation	Key refs
ICS in unselected bronchiectasis	Broad corticosteroid-sensitive airway inflammation	Direct bronchiectasis RCTs	Biological and some symptom/sputum-volume effects were observed, but improvements in exacerbations, lung function and other major clinical outcomes were inconsistent.	No validated biomarker selection in parent trials.	Small older trials; heterogeneous doses/endpoints; conducted before modern endotyping.	Routine long-term ICS is not supported for bronchiectasis without another established indication.	(50, 55, 58, 60)
ICS in eosinophilic subgroups	Potential corticosteroid-responsive eosinophilic inflammation	Post-hoc RCT analyses and observational cohorts	Some fluticasone/pool and large real-world analyses suggest fewer exacerbations in patients with higher eosinophils; a budesonide reanalysis did not reproduce the signal.	Higher BEC may act as a candidate effect modifier.	Post-hoc analyses, reuse of parent datasets, observational confounding and data-derived thresholds.	BEC is not yet a validated predictive biomarker for ICS response.	(14–16, 49, 59)
ICS safety	Potential local immunosuppression in chronically infected airways	Observational bronchiectasis and mixed-airway-disease evidence	Associations have been reported with respiratory infection and pulmonary NTM disease; pneumonia data are influenced by COPD overlap.	No validated eosinophil threshold defining net benefit vs. harm.	Non-randomized safety evidence and residual confounding.	Any putative eosinophil-associated benefit must be weighed against infection risk.	(61–63)
Benralizumab (MAHALE)	IL-5R*α*; depletion of eosinophils	Direct bronchiectasis phase 3 randomized placebo-controlled trial	Near-complete blood/airway eosinophil depletion occurred, but no demonstrated reduction in annualized exacerbations or clear improvement in symptoms, FEV1 or quality of life.	Only a small minority of randomized participants had BEC ≥300 cells/µL.	Severe under-recruitment relative to the intended sample and inadequate enrichment for persistent eosinophilic biology.	Target engagement without demonstrated clinical efficacy; the trial does not validate an actionable eosinophilic endotype.	(17)
Mepolizumab/benralizumab in severe eosinophilic asthma with bronchiectasis	IL-5/IL-5Rα	Indirect real-world, registry and small prospective asthma studies	Asthma control, asthma exacerbations and corticosteroid requirements improved in treated severe eosinophilic asthma populations; mucus-plug improvement has also been reported.	Patients were selected for severe eosinophilic asthma rather than bronchiectasis-specific T2 biology.	Bronchiectasis-specific randomized endpoints generally absent.	Supports biologic treatment when severe eosinophilic asthma independently meets established indications; does not establish bronchiectasis-specific efficacy.	(65–68, 70)
Dupilumab	IL-4Rα blockade of IL-4/IL-13 signaling	Predominantly indirect severe-asthma evidence with coexisting bronchiectasis	Clinical improvement has been reported for severe type 2 asthma in patients who also have bronchiectasis.	Asthma-derived T2 selection.	No adequate direct bronchiectasis efficacy trial.	Treat according to established comorbid disease indications; bronchiectasis-specific efficacy remains unproven.	(69)
Anti-TSLP/upstream T2 blockade	TSLP and upstream epithelial type 2 signaling	Mechanistic rationale largely extrapolated from asthma	No direct therapeutic trial has established bronchiectasis-specific efficacy.	Potentially broader than BEC-only selection, but no validated bronchiectasis biomarker strategy.	Bronchiectasis-specific pathway validation and therapeutic trials are lacking.	Investigational; should not be extrapolated from asthma efficacy.	(86, 87)

Evidence interpretation distinguishes direct bronchiectasis trials from studies in asthma/bronchiectasis overlap populations. A prognostic biomarker is associated with outcome irrespective of treatment; a predictive biomarker identifies differential treatment effect. Current evidence supports BEC only as a candidate effect modifier, not a validated predictive biomarker. BEC, blood eosinophil count; FEV1, forced expiratory volume in 1 s; ICS, inhaled corticosteroid; IL, interleukin; NTM, nontuberculous mycobacteria; RCT, randomized controlled trial; TSLP, thymic stromal lymphopoietin.

## 10 From phenotype to endotype-informed management

The current evidence does not support a standalone clinical algorithm for “type 2 bronchiectasis”. A more defensible approach is to integrate eosinophilic biomarkers into established bronchiectasis assessment while distinguishing guideline-supported practice from a proposed research framework for endotype-informed management (76–78).

The first step is systematic characterization of conditions capable of generating the same biomarkers. Asthma is common in bronchiectasis and is associated with greater eosinophilia, rhinosinusitis, nasal polyposis, and Aspergillus sensitisation (79). ABPA and Aspergillus sensitisation are additional major sources of type 2-associated inflammation (9, 45), while CRSwNP and COPD overlap can also influence biomarker interpretation and corticosteroid decisions (80). Severe-asthma studies demonstrate particularly strong eosinophilic airway activation in patients who also have bronchiectasis, underscoring why such populations cannot be generalized to bronchiectasis alone (81).

Current ERS guidance incorporates blood eosinophils, total IgE, and Aspergillus-specific testing into etiological assessment, while the 2026 CHEST guideline provides complementary evidence-based recommendations for adult bronchiectasis management and emphasizes the potential value of accurate phenotyping and endotyping. Revised ISHAM guidance provides the diagnostic framework for distinguishing ABPA from other bronchiectasis phenotypes (60, 82, 83). These tests are clinically important, but their primary current role is etiological characterization rather than assignment to a treatment-defined endotype.

For patients without an obvious alternative explanation for eosinophilia, repeated stable-state BEC is a pragmatic next step for research-oriented phenotyping because single measurements are incompletely stable (10, 11). Biomarker interpretation should then be integrated with chronic infection, microbiology, exacerbation history, and disease etiology rather than used in isolation (6, 9, 28, 30, 32).

A useful conceptual framework can distinguish predominantly neutrophilic/infection-associated disease, an eosinophilic phenotype, mixed inflammatory disease, and a candidate type 2 endotype. These are organizing categories, not validated diagnostic classes. Assignment to a candidate endotype would ideally require reproducible systemic evidence, airway biological coherence, and careful attribution of asthma, ABPA, fungal sensitisation, CRS, and COPD. More detailed airway or molecular profiling remains primarily a research tool.

Treatment should remain anchored to existing evidence. Standard bronchiectasis therapy applies regardless of eosinophil status. ICS should be used when there is an established indication such as asthma or COPD rather than on the basis of BEC alone. Biologics should likewise be prescribed according to approved indications for severe asthma, ABPA, or other type 2 diseases until bronchiectasis-specific efficacy is demonstrated (84, 85).

Reassessment is intrinsic to this framework because both stable-state BEC and exacerbation endotypes can vary over time (10, 11, 33). The present transition toward precision medicine should therefore be understood as a shift in methodology and trial design, not as an established therapeutic state (Figure 3).

**Figure 3:**
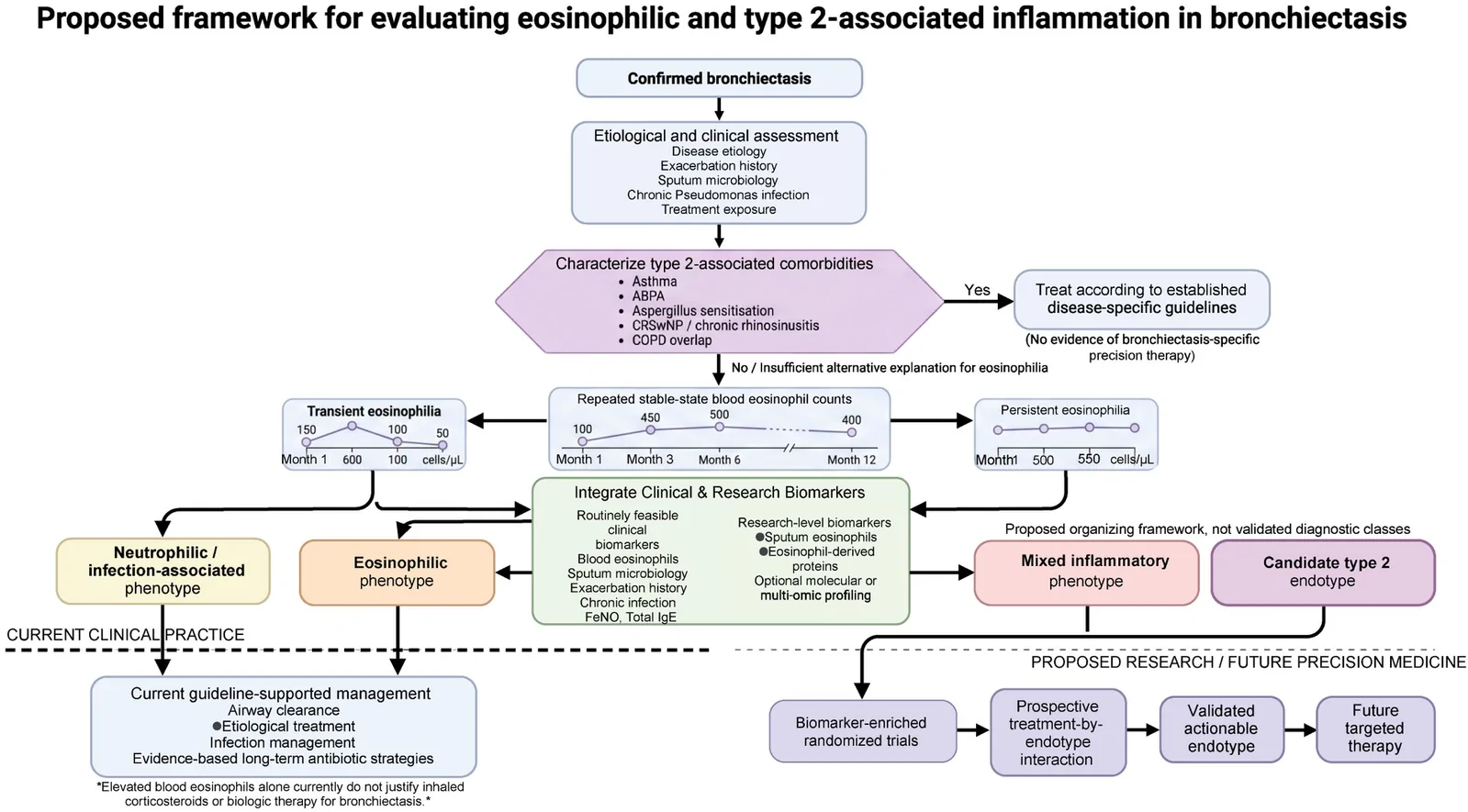
Proposed framework for evaluating eosinophilic and type 2-associated inflammation in bronchiectasis. The framework separates current clinical assessment from a proposed research pathway for endotype validation. Current management remains based on established bronchiectasis care and recognized comorbid indications, whereas repeated phenotyping, airway biological confirmation, and biomarker-enriched treatment studies represent proposed steps toward future endotype-informed therapy.

## 11 Discussion

The evidence reviewed here supports eosinophilic inflammation as a reproducible component of bronchiectasis heterogeneity, but it does not yet support a unified, bronchiectasis-specific type 2 endotype that can be used to guide treatment. This distinction is central to interpreting the rapidly expanding literature. Blood eosinophilia, airway eosinophilia, type 2-associated biomarker profiles, mechanistically coherent endotypes, and predictive treatment-response subgroups represent progressively stronger levels of biological inference rather than interchangeable labels. Large multicohort and airway-focused studies now provide convincing evidence that eosinophilic inflammation can occur in bronchiectasis independently of overt asthma or active ABPA, while direct sputum and proteomic analyses demonstrate eosinophil activation within bronchiectatic airways (6–8). However, the modest concordance between circulating and airway eosinophilic measures, discordance among BEC, FeNO and IgE, and incomplete temporal stability of BEC indicate that a single peripheral biomarker cannot adequately define the underlying airway mechanism (8, 10–13). The current evidence therefore positions BEC most appropriately as a pragmatic stratification marker rather than a validated surrogate of airway type 2 biology or a predictive biomarker.

Several apparent contradictions in the literature become more coherent when bronchiectasis inflammation is viewed as multidimensional and dynamic rather than as a binary neutrophilic-vs.-eosinophilic classification. Molecular studies identify mixed neutrophilic/type 2-associated inflammatory states, and eosinophilic signals can coexist with chronic infection and Pseudomonas-dominated microbiota (8, 30, 31). In parallel, longitudinal observations show that inflammatory phenotypes may vary over time and between stable disease and exacerbations (10, 11, 33). This framework may also explain the apparently divergent prognostic associations of eosinophils. Persistent or higher eosinophilia has been associated with increased exacerbation susceptibility in some populations, whereas low BEC or eosinopenia has been associated with greater disease severity, hospitalization, and mortality in others (6, 11, 37, 38, 53, 54). These observations should not be interpreted as evidence for opposite causal effects of eosinophils. Rather, eosinophil measurements may identify biologically distinct disease states whose clinical significance depends on persistence, infection burden, treatment exposure, disease severity, and the compartment in which inflammation is measured. Accordingly, the prognostic value of BEC is likely to be context dependent rather than linear.

The therapeutic literature provides the most stringent test of whether eosinophilic phenotyping has progressed to clinically actionable endotyping. Historical ICS trials demonstrate that corticosteroids can modify airway inflammatory indices or selected symptoms, but their effects on exacerbations, lung function, and other major outcomes have been inconsistent. Subsequent *post-hoc* and observational analyses suggest that higher BEC may identify patients with greater corticosteroid responsiveness, although this signal has not been consistently reproduced and remains vulnerable to retrospective threshold selection and confounding (14–16, 49). BEC should therefore be regarded as a candidate effect modifier rather than a validated predictive biomarker. MAHALE provides an even more informative example of the distinction between biological plausibility and therapeutic validation. Benralizumab achieved profound eosinophil depletion but did not demonstrate improvement in major clinical outcomes in the population studied (17). Severe under-recruitment and limited enrichment for persistent eosinophilic disease restrict inference about a rigorously selected eosinophilic subgroup, but the conceptual lesson remains important: pharmacological target engagement does not establish that the targeted pathway is disease-driving, nor does it validate an endotype or demonstrate clinical efficacy. Similarly, favorable responses to mepolizumab, benralizumab or dupilumab in severe asthma complicated by bronchiectasis remain evidence for treating the coexisting type 2 airway disease, not proof of bronchiectasis-specific benefit (65–70).

Progress toward an actionable endotype therefore requires a higher evidentiary threshold than phenotype recognition alone. A candidate bronchiectasis-specific type 2 endotype should first demonstrate temporal reproducibility and biological coherence across systemic and airway compartments. Major competing sources of type 2-associated biomarkers—including asthma, ABPA, Aspergillus sensitisation, chronic rhinosinusitis with or without nasal polyposis, COPD overlap, and corticosteroid exposure—must then be prospectively characterized rather than treated as residual confounding (9, 45, 79). Microbiological context should also be incorporated because chronic infection and inflammatory phenotype are overlapping dimensions rather than mutually exclusive processes. Molecular and proteomic classifiers may ultimately provide greater mechanistic specificity than BEC alone, but they currently remain dependent on platform, cohort, and sampling context and require external and longitudinal validation (8, 30, 33). The decisive step is prospective demonstration that the proposed endotype modifies treatment effect. Prognostic association, biological plausibility, and even complete target suppression are insufficient unless a prespecified biomarker or endotype identifies differential clinical benefit from therapy. Together, these requirements define a proposed evidence hierarchy from eosinophilic phenotype recognition to a clinically actionable bronchiectasis endotype (Figure 4).

**Figure 4:**
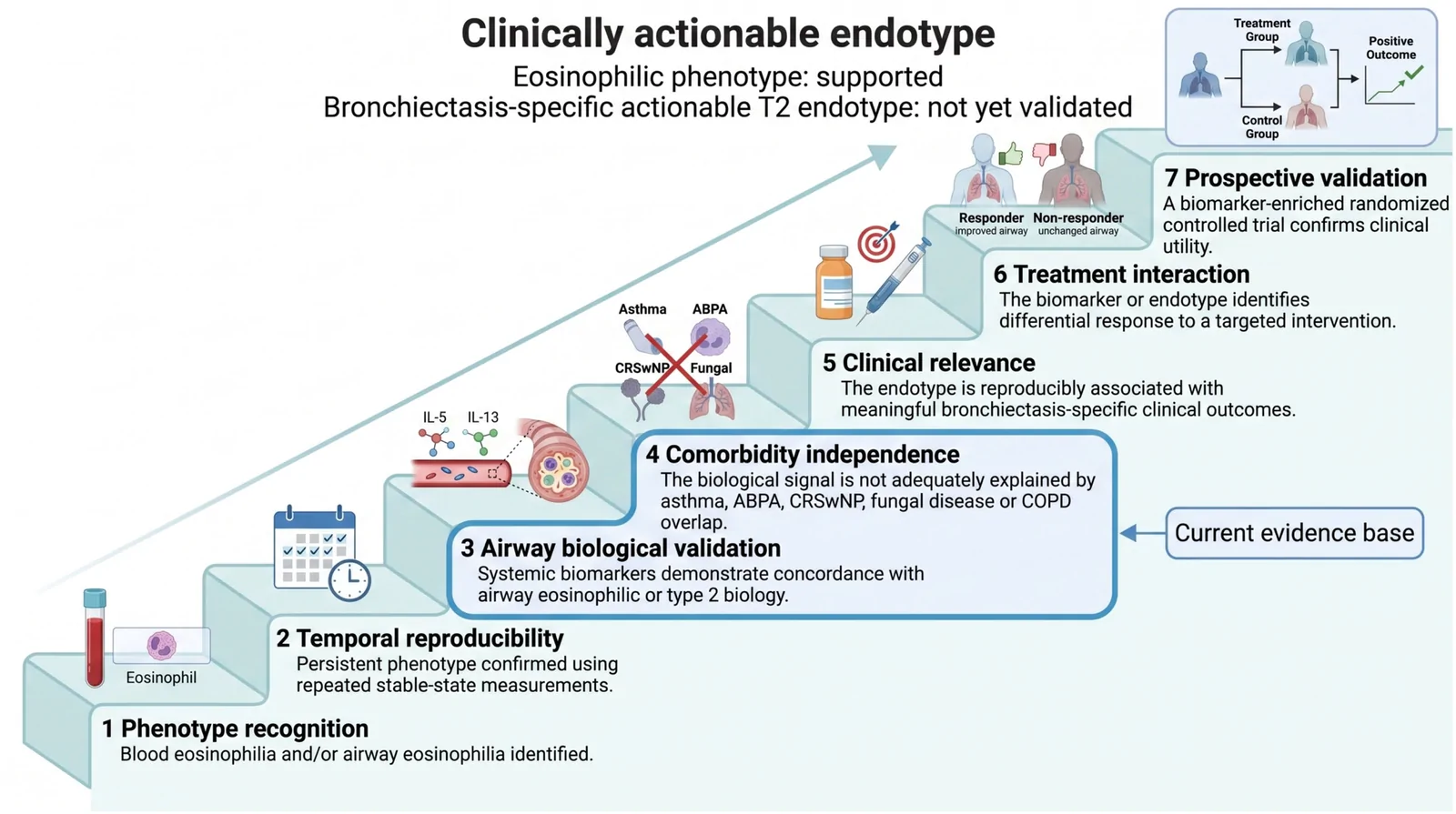
Evidence required to translate an eosinophilic phenotype into an actionable bronchiectasis endotype. The proposed hierarchy progresses from phenotype recognition through temporal reproducibility, airway biological validation, control of major type 2-associated confounders, clinical relevance, treatment interaction, and prospective biomarker-enriched validation. Pharmacological target engagement alone is insufficient to establish a clinically actionable endotype.

Future studies should therefore be designed around endotype validation rather than retrospective biomarker subdivision. Repeated stable-state BEC measurements should be combined, where feasible, with paired airway eosinophil counts, eosinophil-derived proteins, or pathway-level markers to determine whether airway confirmation improves specificity beyond blood phenotyping alone. Biomarker-enriched trials should prespecify eosinophilic persistence, characterize major type 2 comorbidities, stratify relevant microbiological states, and use bronchiectasis-specific outcomes including exacerbations, hospitalization, symptoms, quality of life, and lung-function trajectories. Most importantly, treatment-by-biomarker interactions should be defined prospectively so that predictive utility can be distinguished from simple prognostic association. The field has therefore moved beyond asking whether eosinophilic inflammation exists in bronchiectasis. The next step is to determine whether a reproducible, airway-coherent and comorbidity-independent inflammatory state can prospectively identify patients who derive differential benefit from targeted therapy. Until that standard is met, eosinophilic phenotyping should inform biological investigation and trial enrichment rather than constitute a standalone treatment algorithm.

## 12 Conclusion

Eosinophilic inflammation is now a reproducible component of bronchiectasis heterogeneity. Multiple cohorts, including studies that exclude overt asthma and active ABPA, identify a meaningful eosinophilic subgroup, while airway studies confirm direct eosinophil activation within bronchiectatic airways (6–8). However, peripheral eosinophilia, airway eosinophilia, and type 2 inflammation remain only partially overlapping constructs, and their interpretation is influenced by temporal variability and by asthma, ABPA, fungal sensitisation, CRS, and COPD.

The evidence is therefore stronger for eosinophilic phenotyping than for a unified bronchiectasis-specific type 2 endotype. BEC is the most clinically feasible stratification marker, but no single biomarker has sufficient airway specificity, reproducibility, and prospective treatment-predictive value to guide routine endotype-directed therapy. ICS response signals remain exploratory, and MAHALE showed that profound eosinophil depletion does not necessarily translate into clinical benefit in a broadly selected population (16, 17).

Type 2-targeted treatment should remain investigational for bronchiectasis itself unless independently indicated for a coexisting disease. The field should now move from single-marker eosinophilic phenotyping to longitudinal, airway-validated, and prospectively treatment-tested endotyping, using biomarker-enriched randomized trials to establish whether any subgroup derives differential bronchiectasis-specific benefit.
